# Genetic and economic benefits of foreign sire contributions to a domestic sheep industry; including an Ireland-New Zealand case study

**DOI:** 10.1186/s12711-020-00594-y

**Published:** 2021-01-06

**Authors:** Nicola Fetherstone, Fiona S. Hely, Noirín McHugh, Fiona M. McGovern, Peter R. Amer

**Affiliations:** 1 Teagasc, Animal and Grassland Research and Innovation Centre, Mellows Campus, Athenry, Co. Galway, Ireland; 2 School of Agriculture and Food Science, University College Dublin, Belfield, Dublin 4, Ireland; 3 AbacusBio International, Public Trust Building, 442 Moray Place, Dunedin, 9016 New Zealand

## Abstract

**Background:**

Importation of foreign genetics is a widely used genetic improvement strategy. However, even if the foreign genetic merit is currently greater than the domestic genetic merit, differences in foreign and domestic trends mean that the long-term competitiveness of an importation strategy cannot be guaranteed. Gene flow models are used to quantify the impact that a specific subpopulation, such as foreign genetics, can have over time on the genetic or economic benefit of a domestic industry.

**Methods:**

We used a deterministic recursive gene flow model to predict the commercial performance of lambs born across various subpopulations. Numerous breeding strategies were evaluated by varying market share, proportions of rams selected for mating, genetic trend, superiority of foreign genetics over domestic genetics and frequency of importation. Specifically, an Ireland-New Zealand case study was simulated to quantify the potential gain that could be made by using foreign sire contributions (New Zealand) in a domestic sheep industry (Ireland).

**Results:**

Genetic and economic gains were generated from alternative breeding strategies. The ‘base scenario’ (i.e. representing the current industry) predicted an average genetic merit value of €2.51 for lambs born and an annualised cumulative benefit of €45 million (m) after 20 years. Maximum genetic (€9.45 for lambs born) and economic (annualised cumulative benefit of €180 m after 20 years) benefits were achieved by implementing the ‘PRO-intense-market scenario’ which involved shifting market share away from conservative domestic breeders and reducing the proportion of rams that were selected for mating by progressive domestic breeders from the top 40% to the top 20%, without the use of any foreign genetics. The ‘PROFOR scenario’, which considered the use of foreign and progressive domestic genetics, predicted an average genetic merit value of €7.37 for lambs born and an annualised cumulative benefit of €144 m, after 20 years.

**Conclusions:**

Our results demonstrate that there is opportunity for a domestic industry to increase industry benefits without the use of foreign genetics but through an attempt to shift the market share away from conservative domestic breeders towards progressive domestic breeders. However, the importation and use of progressive foreign genetics may be an effective method to trigger a change in behaviour of conservative domestic breeders towards the use of progressive genetics.

## Background

Within a breeding pyramid, subpopulations can be stratified into various tiers across multiple species such as sheep, beef and dairy cattle [1–3], by using a concept that was originally introduced many decades ago by Bichard [4]. Each subpopulation or tier contributes differently to the rates of genetic and economic benefits achieved [5]. Multi-tiered breeding structures are common practise in sheep breeding populations including Ireland, New Zealand and Australia [1, 6, 7]. Specifically within sheep industries, two tiered structures that include nucleus (ram breeder) and commercial tiers or three tiered structures that also include a multiplier tier are used [5]. Using various subpopulations, a gene flow model can be generated to identify optimal breeding strategies that could maximise the economic and genetic benefits.

Gene flow models are also useful to account for the time-lag involved when transitioning from one breeding strategy to another [1–3]. Unlike steady state models as introduced by Rendel and Robertson [8], deterministic gene flow models allow for the gradual improvement in the rate of genetic gain achieved. Previously, gene flow models have been used in the sheep, beef and dairy cattle industries to quantify the impact of performance recording, genomic selection, genetic evaluations or the use of foreign genetics on genetic improvement [1, 2, 9].

Importation and the use of superior foreign genetics, as an alternative to domestic genetics, is a strategy that has been used to accelerate the domestic genetic gain that can be achieved [10, 11]. However, although foreign genetics have often been proven to be genetically superior to domestic genetics [12], their long-term suitability depends on the consistency in the breeding goal and trait improvement trajectory for the foreign versus domestic genetics. Thus, trade-offs between short- and long-term impacts of importation strategies are complex. This paper focuses on the methodology required to address this in a national sheep breeding programme through the importation of foreign genetics from New Zealand to the domestic Irish industry. The model generates genetic and economic benefit values that demonstrate the suitability of foreign sires for long-term use within the Irish domestic sheep industry. If suitable, an optimal breeding programme can then be developed and implemented nationally in an attempt to maximise genetic gain within the industry.

## Methods

### Overview of the model

A gene flow model with multiple flows of predicted genetic merit across subpopulations of a sheep industry was created using Microsoft Excel. The model predicted the genetic improvement in overall economic merit of future generations of commercial sheep as a consequence of the incorporation or exclusion of foreign genetics in a proportion of ram breeder flocks. The model also assessed the impact of the implementation of various breeding strategies using multiple scenarios (described below). Depending on the scenario under investigation, the market share of each subpopulation varied on an annual basis. Similarly, for each subpopulation, the age structure of the ram and ewe population, genetic trends, genetic merit,  the proportion of rams selected for mating and selection differentials could also be varied. Discount factors and the level of superiority of foreign sires over domestic sires, as well as the size of the national ewe population and weaning rate, which ultimately determined the number of lambs born alive per year, could be adjusted within each modelled scenario.

Annualised cumulative benefits were calculated to quantify and compare the impact of each alternative breeding strategy investigated. The final output of the gene flow model was the predicted average genetic merit value of commercial lambs born during a given year, which were then aggregated to industry-level benefits and expressed as a cumulative value. The model could be refined to suit any species or any country. To start, the model generated predictions for the genetic merit of animals within a number of subcategories including: ewes, rams born, rams mated and lambs born during a historic phase from the year −10 to year 0. This historic phase was identical for every strategy and reflected the current genetic merit of subpopulations and their recent genetic trends. Year 0 was described as the base year for the model. Thereafter, a recursive predictive phase was generated from year 1 onwards. This predictive phase modelled future gene flow and genetic trends in accordance with each breeding strategy scenario. Standard partial budgeting techniques were used to aggregate the benefits into present values, with a focus on the overall industry impact after a certain time horizon.

### Model creation: historic phase

The historic phase was classified as year −10 up to and including year 0. The genetic merit of animals in each subpopulation at year 0 (i.e. $Y=0$), was derived using available industry data. Historic levels of genetic merit (i.e. from $-10\le Y\ge -1$) for each subpopulation were back-calculated based on recent genetic trends.

#### Ewes

At year 0, the average genetic merit value of ewes mated for each subpopulation $$W$$, denoted as ($${G}_{W, Y=0}^{Ewes })$$, were specified using available industry data.

#### Rams born

At year 0, the average genetic merit value, at birth, of selected breeding rams within each subpopulation $W$, denoted as $\left( {G_{W,Y = 0}^{Rams\;born} } \right)$, was calculated as:

1 $$G_{W, Y = 0}^{Rams\;born} = G_{W, Y = 0}^{Ewes} + \Delta G_{W, Y \le 0} \left( {\overline{X}_{W, Y = 0}^{Rams} + 2\cdot\overline{X}_{W, Y = 0}^{Ewes} } \right),$$

where ($${G}_{W, Y=0}^{ewes})$$ is the average genetic merit value of the breeding ewes in the subpopulation $$W$$ at year $$Y=0$$, ($${\Delta G}_{W, Y\le 0}$$) is the annual genetic trend used to adjust the genetic lag associated with rams during the historic phase, $$\left( {\overline{X}_{W, Y = 0}^{Rams\;born} } \right)$$  and $$\left( {\overline{X}_{W, Y = 0}^{Ewes} } \right)$$ are the average age of the rams and ewes in the subpopulation $$W$$ at the time when their progeny are born (in years). The derivation of Eq. 1 is described in Additional file 1.

#### Rams mated

At year 0, the average genetic merit value of rams used for mating, denoted as $\left( {G_{W,Y = 0}^{Rams\;mated} } \right)$, was calculated as:

2 $$G_{W,Y = 0}^{Rams\;mated} = G_{W,Y = 0}^{Rams\;born} - \left( {\overline{X}_{W,Y = 0}^{Rams\;born} \cdot \Delta G_{W,Y \le 0} } \right),$$

where $\left( {G_{W,Y = 0}^{Rams\;born} } \right)$ is the average genetic merit value of rams born in year 0, $\left( {\overline{X}_{W, Y = 0}^{Rams\;born} } \right)$ is the average age (in years) of rams at mating, and (${\Delta G}_{W, Y\le 0})$ is the recently observed genetic trend for all animals in subpopulation $W$.

### Model creation: recursive phase

From year 1 onwards (i.e. $Y\ge 1$), genetic trends of the ewes, rams born, rams mated and lambs born were calculated recursively.

#### Ewes

Beyond the historic phase, the average genetic merit value of ewes, denoted as (${G}_{W, Y\ge 1}^{Ewes}),$ was calculated, assuming no selection among female replacements, as:

3 $$G_{W, Y \ge 1}^{Ewes} = \mathop \sum \limits_{i = 1}^{6} \left( {G_{W, Y - i}^{Lambs} \cdot p_{W, i}^{Ewes} } \right),$$

where $Y$ is the year the ewes are mated, $({G}_{W, Y-i}^{Lambs})$ is the average genetic merit value of the lambs born $i$ years before year $Y$ within a subpopulation $W$ and ${(p}_{W, i}^{Ewes})$ is the proportion of breeding ewes within the given age group $(i)$ of a specific subpopulation $W$.

#### Rams born

Beyond the historic phase, the average genetic merit values, at birth, of selected breeding rams within each subpopulation $W$, denoted as $\left( {G_{W, Y \ge 1}^{Rams\;born} } \right)$, were calculated as:

4 $$G_{W,Y \ge 1}^{Rams\;born} = \left( {0.5 \cdot G_{W, Y \ge 1}^{Ewes} ) + (0.5 \cdot G_{W,Y \ge 1}^{Rams\;Mated} } \right) + S.Diff_{W}^{Rams\;born},$$

where $$\left( {G_{W, Y \ge 1}^{Ewes} } \right)$$) and $$\left( {G_{W, Y \ge 1}^{Rams\;mated} } \right)$$ are the average genetic merit values of the ewes and of the rams mated to those ewes, respectively. The selection differential of the rams born, denoted as ($${S.Diff}_{W})$$, accounted for the difference in the average genetic merit value of all possible candidate ram lambs and those actually retained for breeding.

#### Rams mated

Beyond the historic phase, the average genetic merit value of rams used for mating, denoted as $\left( {G_{W, Y \ge 1}^{Rams\;mated} } \right)$, were calculated as:

5 $$G_{W, Y \ge 1}^{Rams\;mated} = \mathop \sum \limits_{J = 1}^{s} \left[\tau_{J,W} \cdot \mathop \sum \limits_{i = 1}^{6} \left(G_{W, Y - i}^{Rams\;born} \cdot p_{W, i}^{Rams\;mated} \right)\right],$$

where $Y$ is the year the rams are mated, ${\tau }_{J,W}$ is the proportion of rams sourced by subpopulation $W$ from $s$ number of alternative subpopulations of rams (indexed $J$) that are used for breeding purposes and ${p}_{W, i}^{Rams\;mated}$ is the proportion of rams mated within a subpopulation $W$ of a given age group $(i)$.

#### Lambs born

All average genetic merit values of lambs born, denoted as $\left( {G_{W, Y}^{Lambs\;born} } \right)$, were calculated regardless of year ($-10\le Y\ge 20$) using:

6 $$G_{W, Y}^{Lambs\;born} = (0.5 \cdot G_{W, Y}^{Rams\;mated} ) + \left( {0.5 \cdot G_{W, Y}^{Ewes } } \right),$$

where ($${G}_{W, Y}^{Rams\;mated})$$ is the average genetic merit value of the rams mated and ($${G}_{W, Y}^{Ewes})$$ is the average genetic merit value of the ewes mated during year $$Y$$.

### Investigation of industry benefits

The value of genetics to the industry per commercial lamb born in year $Y$, denoted as ${(V}_{W=COM, Y}^{Lambs\;born})$, was determined using:

7 $$V_{W = COM, Y}^{Lambs\;born} = \beta \left( {G_{W = COM, Y > 0}^{Lambs\;born} - G_{W = COM, Y = 0}^{Lambs\;born} } \right),$$

where $\beta$ translates the average genetic merit value into gross margin benefit to farmers, and has a value of 1 where the units of measurement for the genetic merit of trait $G$ is the same as units of measurement for the index value (i.e. monetary value per lamb born).

#### Undiscounted annual benefit

The total annual income (€ millions) generated by the industry in year $Y$ as a result of the commercial industry adopting a particular scenario, denoted as $\left({AB}_{Y}\right)$, was calculated as:

8 $$AB_{Y} = N_{Y} \cdot\left( {\frac{{ V_{W = COM, Y }^{Lambs\;born} }}{1,000,000}} \right),$$

where ${N}_{Y}$ is the number of lambs born in year $Y$, predicted using available industry data on the national ewe population size and weaning rates. This could be modified to demonstrate any change in the size of the industry.

#### Discounted annual benefit

The total annual income (€ millions) generated by the industry in year $Y$ was discounted to account for the change in value of money over time $({DAB}_{ Y}$), and was calculated as:

9 $$DAB_{ Y} = AB_{Y} \cdot DF_{Y}.$$

The discount factor $(DF)$ in year $Y$ was based on inflation and mortgage prices with the discount rate equal to 5%:

10 $$DF_{Y} = \left( {\frac{1}{1 + 0.05}} \right)^{Y}.$$

#### Discounted cumulative benefit

The total income generated over a specific period of time was evaluated, while accounting for the lag between selection decisions and the flow of genetic contributions. The discounted cumulative benefit, denoted as ($${DCB}_{Y })$$ was calculated as:

11 $$DCB_{Y } = \mathop \sum \limits_{i = 1}^{Y} DAB.$$

#### Additional five-year benefit

As genetic gain is permanent and cumulative, the values were augmented in each scenario by assuming that the genetic merit would be sustained for a further 5 years beyond year $Y$, denoted as $(DPB5)$, and was calculated as:

12 $$DPB5_{Y} = AB_{Y} \cdot\mathop \sum \limits_{i = Y + 1}^{Y + 5} (DF_{i} ).$$

#### Present value of the benefits

The present value of the benefits, denoted as (${PVB}_{Y}$), is an overall indicator of the total potential economic benefit that could be gained by the industry as a result of implementation of a given scenario after a certain period of time, while also accounting for the change in the value of money over time.

13 $$PVB_{Y } = DCB_{Y } + DPB5_{Y} .$$

#### Annualised cumulative benefit

The annualised cumulative benefit, denoted as ($${ACB}_{Y }),$$ is an overall indicator of the actual potential economic benefit that could be gained by the industry on an annual basis as a result of implementation of a given scenario after a certain period of time, while also accounting for the change in the value of money over time and was calculated using:

14 $$ACB_{Y } = \frac{{ DCB_{Y } + DPB5_{Y} { }}}{{\mathop \sum \nolimits_{i = 1}^{Y} DF}}.$$

### An Ireland—New Zealand case study

An Ireland-New Zealand case study was undertaken to demonstrate the simulation of the gene flow model. The current Irish sheep industry is based on a two-tiered breeding pyramid with pedigree breeders supplying rams directly to commercial flocks. These pedigree breeders can be subdivided into two groups; those engaged with the replacement index over the last number of years (the top 40 flocks achieving genetic gain), and a group representing those not engaged (all other flocks that failed to achieve genetic progress in the replacement index), with the national performance recording system operated by Sheep Ireland [13]. The gene flow model described above was used to quantify the genetic and economic impacts on the Irish sheep industry of sourcing superior domestic or foreign genetics from Ireland or New Zealand, respectively. Two maternal breeding indexes were incorporated into the gene flow model: the Irish €uro-star Replacement Index and the New Zealand Maternal Worth (NZMW), with breeding traits expressed as estimated breeding values in both indexes. Both indexes are expressed in monetary terms and consist of a range of similar traits aimed at identifying animals with superior maternal genetics as outlined by Santos et al. [14].

The subpopulations modelled represent the Irish sheep industry and are described in Table 1.

**Table 1:** Subpopulations included in the model which represent the Irish sheep industry

Subpopulation	Abbreviation	Description
Ram breeder flocks		
Conservative breeders	CON	Flocks that do not invest in superior genetics and are not achieving genetic gain
Progressive breeders using domestic genetics	PRO	Flocks that are currently ranked in the top 40 flocks for genetic gain but never use foreign genetics
Progressive breeders using foreign genetics	PROFOR	Flocks using foreign genetics via artificial insemination (AI) but also source domestic rams within their own subpopulation
Foreign breeders	FOR	Foreign flocks who supply rams directly to domestic flocks
End-users		
Commercial farmers	COM	Commercial flocks sourcing rams or semen from ram breeder flocks

The average genetic merit value of rams born within the FOR subpopulation in year 0 of each scenario (i.e. where $Y=0$), denoted as (${G}_{FOR, Y=0}^{Rams\;born})$, was calculated as:

15 $$G_{FOR, Y = 0}^{Rams\;born} = G_{PRO, Y = 0}^{Rams\;born} + FORdev,$$

where ${G}_{PRO, Y=0}^{Rams\;born}$ is the average genetic merit value of rams born in the progressive subpopulation (PRO) and $FORdev$ is the adjustment to reflect the superiority of the foreign rams (FOR) based on results from a previous study [12], in comparison to those in the domestic market (explained below).

Over the period of each scenario, the average genetic merit value of FOR rams born, ${G}_{FOR,Y\ge 0}^{Rams\;born}$ was specified in blocks, depending on the frequency of importation (e.g. every 5 or 10 years). Therefore, the average genetic merit value of FOR rams is static for a number of years until another shipment is imported into the domestic market. Incremental jumps in average genetic merit values at each new shipment were predicted by multiplying the genetic trend in the FOR subpopulation, translated into Irish €uro-star Replacement Index units, by the number of years between shipments.

Genetic trends for the Irish €uro-star Replacement Index for the progressive (PRO) and commercial (COM) subpopulations were established based on industry data supplied by Sheep Ireland and were calculated as €0.38 and €0.01 per year, respectively. The progressive breeders using foreign genetics subpopulation (PROFOR) were assumed to have the same genetic trend as the PRO subpopulation (i.e. €0.38 per year). The conservative subpopulation (CON) was assumed to make no genetic progress (i.e. €0.00 per year) in the Irish €uro-star Replacement Index, during either the historic or recursive phases of the model.

The relationship between the domestic maternal index (DOM; the Irish €uro-star Replacement Index) and the foreign maternal index (FOR; the New Zealand Maternal Worth), was calculated using genetic evaluations of animals imported into Ireland from progressive New Zealand flocks as part of a production validation study [12]. These animals had records in both the New Zealand and Irish genetic evaluation databases. The average annual genetic trend for the FOR index was converted into the equivalent DOM index, denoted as ${\Delta G}_{DOM\;index}$ using these records as:

16 $$\Delta G_{DOM\;index} = (b_{DOM\;index \cdot FOR\;index} )\cdot\sum\nolimits_{i = 1}^{z} {\frac{\Delta FOR\;index }{z}} ,$$

where ${b}_{DOM\;index, FOR\;index}$ is the regression coefficient calculated between the DOM and FOR maternal indexes, (i.e. 0.214 in the current case study) and $\Delta\;FOR\;index$ is the average annual genetic trend of the FOR maternal index, calculated using trends from $z$ flocks where in this case $z=6$.

The FOR genetic trend was calculated to be €0.18 per year for ewes mated and rams born during the historic phase, using incremental changes in genetic trend for the aforementioned $z$ source flocks, over a 5-year time span. In order to account for the superiority of the FOR subpopulation over domestic subpopulations (based on results from [12]), rams born were awarded a constant adjustment to their level of genetic merit of €2.00, so that when $Y=0$, the rams born within the FOR subpopulation were €2.00 greater than in the PRO subpopulation.

Table 2 outlines the proportion of rams sourced from a subpopulation $(J)$ for mating within each subpopulation $(W)$ for year 0. It was assumed that in year 0, the PRO, PROFOR and FOR subpopulations sourced rams for mating from within their own subpopulation thereby imitating current industry practices. Proportions of rams selected for mating based on genetic merit values, out of all of those born within the CON, PRO, PROFOR, FOR and COM subpopulations, were set at 100, 40, 40, 65 and 100%, respectively, based on the observed superiority of selected ram lambs. It was assumed that ewe replacements were not selected based on their genetic merit value but on phenotypic information.

**Table 2:** Proportion of rams sourced by end-users from each breeding subpopulation in 2019

End-users	Breeding subpopulations				
	CON	PRO	PROFOR	FOR	COM
CON	0.93	0.07	0	0	0
PRO	0	1	0	0	0
PROFOR^a^	0	1 − x_t_	0	x_t_	0
FOR	0	0	0	1	0
COM	0.87	0.13	0	0	0

*CON* conservative breeders, *PRO* progressive breeders, *PROFOR* progressive foreign breeders, *FOR* foreign breeders, *COM* commercial farmers (source: Sheep Ireland)

^a^PROFOR rams were sourced from FOR and PRO flocks at a proportion (x) specific to time *t*

The proportions of ewes and rams mated within each age group and subpopulation are outlined in Tables 3 and 4, respectively. For the PRO, PROFOR and FOR subpopulations, the mean ages of ewes were assumed equal. For the FOR subpopulation, all rams were assigned a ${S.Diff}_{W}$ equal to 0, their genetic merit value at the time of importation was assumed to be known and they were assumed to be 1 year of age when imported.

**Table 3:** Proportion of ewes within each age group (1 to 6+ years) for each subpopulation

Ewe age	Subpopulations				
	CON	PRO	PROFOR	FOR	COM
1	0	0	0	0	0.08
2	0.30	0.25	0.25	0.25	0.23
3	0.26	0.21	0.21	0.21	0.24
4	0.17	0.14	0.14	0.14	0.17
5	0.14	0.12	0.12	0.12	0.13
6+	0.13	0.28	0.28	0.28	0.15

*CON* conservative breeders, *PRO* progressive breeders, *PROFOR* progressive foreign breeders, *FOR* foreign breeders, *COM* commercial farmers (source: Sheep Ireland)

**Table 4:** Proportion of rams within each age group (1 to 6+ years) for each subpopulation

Ram age	Subpopulations				
	CON	PRO	PROFOR	FOR	COM
1	0.29	0.32	0.32	1.00	0.22
2	0.28	0.28	0.28	0.00	0.30
3	0.18	0.16	0.16	0.00	0.21
4	0.11	0.12	0.12	0.00	0.12
5	0.06	0.05	0.05	0.00	0.07
6+	0.08	0.07	0.07	0.00	0.08

*CON* conservative breeders, *PRO* progressive breeders, *PROFOR* progressive foreign breeders, *FOR* foreign breeders, *COM* commercial farmers (source: Sheep Ireland)

The accuracy values associated with the DOM maternal index (i.e. the Irish €uro-star Replacement Index) were retrieved from the Sheep Ireland database for each subpopulation. The accuracy values associated with year 0 were 0.39, 0.45, 0.45, 0.45 and 0.38 for CON, PRO, PROFOR, FOR and COM, respectively. The accuracy values were incorporated into selection differential calculations for rams born within the recursive phase of the model.

The current number of breeding ewes in the Irish sheep population was assumed to be 2,560,180 with a weaning rate of 1.3 lambs per ewe [8], which equates to approximately 3,328,234 lambs born annually. All results are presented using a 20-year planning horizon. Annualised cumulative benefit values are presented relative to the number of lambs born annually (assumed to remain static each year). Genetic merit values are presented as an average of the merit of all the lambs born within a commercial subpopulation in a specific year.

### Scenarios

**Fig. 1:**
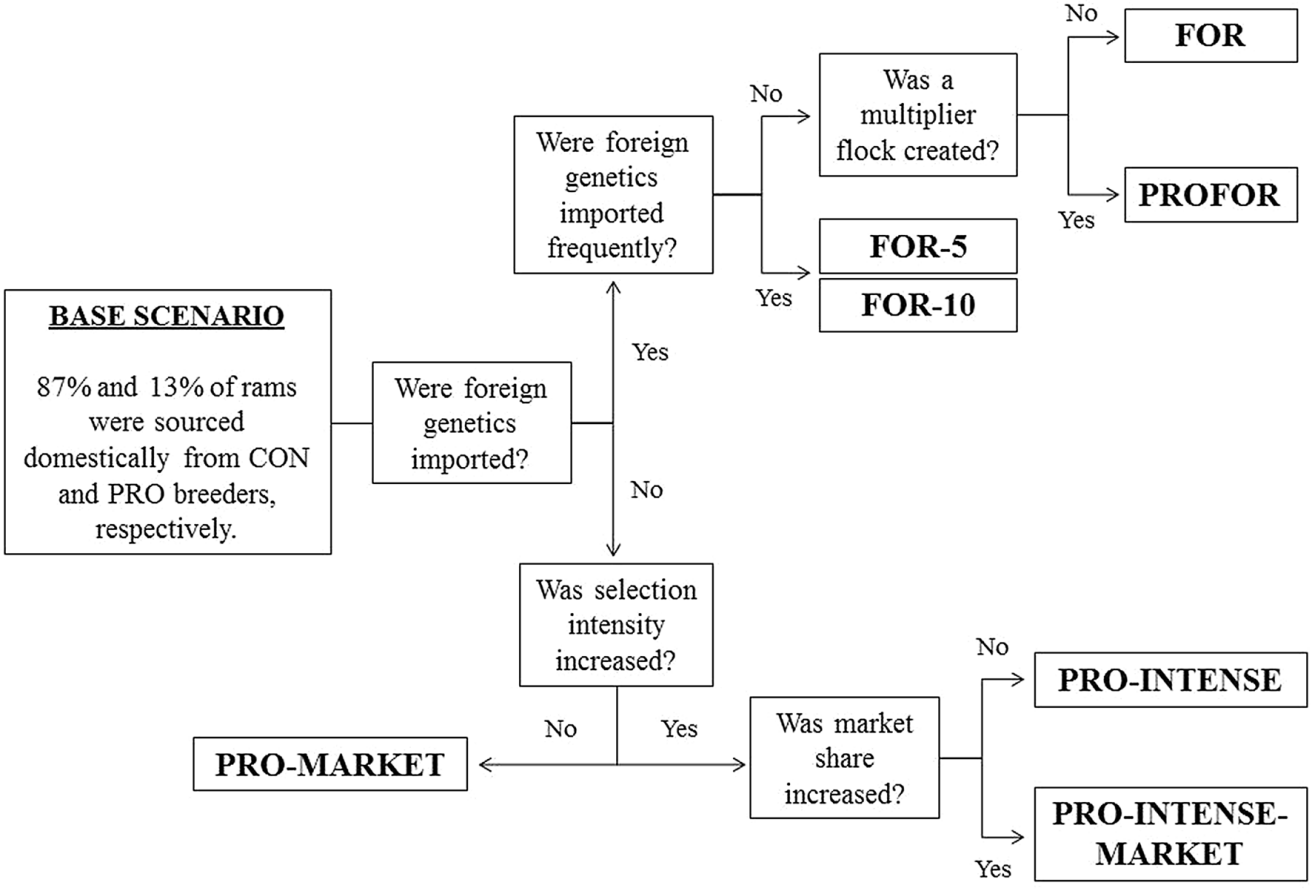
Scheme of possible scenarios for implementation

Several scenarios were investigated to reflect various possible changes within each of the subpopulations and included shifts in market share and/or the proportion of rams selected for mating, as well as variation in the frequency of importation of FOR sires into the domestic industry. A schematic view of possible scenarios for implementation within the industry is in Fig. 1.

The ‘base scenario’ replicated the current Irish industry and predicted future trends where no changes were implemented, with the COM subpopulation continuing to source 87% and 13% of rams from CON and PRO subpopulations, respectively.

A series of scenarios were then undertaken to evaluate the impact of a shift in the market share away from the CON subpopulation towards more progressive domestic or foreign subpopulations (i.e. PRO, PROFOR or FOR).

In the ‘PRO-intense scenario’ a reduction in the proportion of rams selected for mating was investigated, whereby the PRO subpopulation only retained rams for mating that were ranked in the top 20% of rams (instead of from the top 40% as in the base scenario) for genetic merit on the domestic market index. No shift in market share was associated with this scenario.

In the ‘PRO-market scenario’, the impact of increasing the market share of the PRO subpopulation was investigated whereby the market share of the CON subpopulation was reduced by 5% per year of the market share value in the previous year, from year 1 to 20. This market share was transferred to the PRO subpopulation, which thereby increased by 5% of the value in the previous year, per year. The proportion of rams selected for mating was  the same as in the base scenario.

In the ‘PRO-intense-market scenario’, a combination of a 5% shift per year of the market share value in the previous year towards the PRO subpopulation and a reduction in the proportion of rams selected for use at mating was investigated, where only rams that ranked in the top 20% for genetic merit on the domestic market index were retained.

In the ‘PROFOR scenario’, FOR genetics were introduced into the domestic market. From year 1 onwards, market share of the CON subpopulation declined by 5% of the market share value in the previous year, per year; in the first 5 years (i.e. years 1 to 5) this market share was evenly divided between the PRO and FOR subpopulations (i.e. 2.5% increase in market share for both PRO and FOR per year). From year 6 onwards, no FOR subpopulation existed but rather the market share of both the PRO and PROFOR subpopulations grew by 2.5% annually. The PROFOR subpopulation was generated based on sire matings from the PRO and FOR subpopulations within the first 5 years of this scenario.

In the ‘FOR scenario’, the COM subpopulation sourced FOR genetics directly from New Zealand through a single shipment of live rams and/or frozen semen in year 0, with the objective of widespread use via laparoscopic artificial insemination. From year 1 onwards, the market share of the CON subpopulation declined by 5% of the market share value in the previous year, per year. This market share was evenly divided between the PRO and the FOR subpopulations. Two sub-scenarios within the FOR scenario were also investigated, i.e. a ‘FOR-5 scenario’ and a ‘FOR-10 scenario’ that followed similar trends for market share shifts as described in the FOR scenario, however new shipments of FOR genetics were imported every 5 or 10 years, respectively.

## Results

### Mean age and genetic trends

**Fig. 2:**
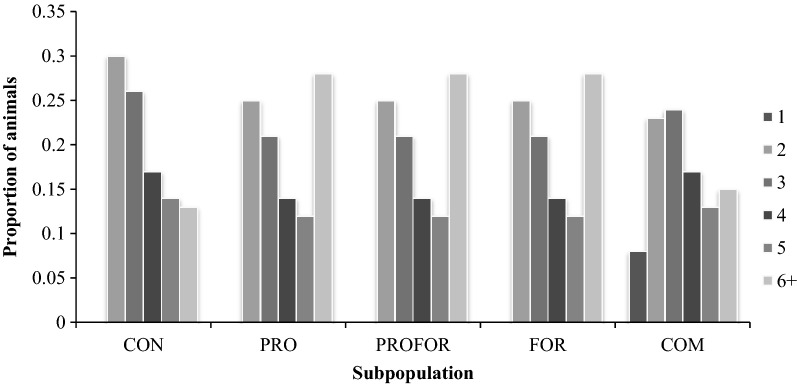
Proportion of ewes at different ages within each subpopulation. The proportion of animals across each subpopulation has a total value of 1. Source: Sheep Ireland. Subpopulations: *CON* conservative breeders, *PRO* progressive breeders, *PROFOR* progressive foreign breeders, *FOR* foreign breeders, *COM* commercial farmers

The mean age of rams and ewes mated within each subpopulation, based on data sourced from the Sheep Ireland database are in Table 5. The mean ewe age for the PRO, PROFOR and FOR subpopulations was 3.97, and was 0.43 and 0.48 years older than that of the CON and COM subpopulations, respectively. Regardless of genetic merit status, all groups of pedigree ewes (i.e. subpopulations in the CON, PRO, PROFOR and FOR) were on average older than those in the COM subpopulation (average 3.86 vs. 3.49 years). The age structure of COM ewes was normally distributed in comparison to ewes within the pedigree subpopulations, which was skewed, demonstrating a younger flock and therefore, a higher replacement rate (Fig. 2). An additional 10% of ewes survived for more than 4 years of age in the PRO subpopulation compared to the CON subpopulation. The age distribution of rams was similar across all subpopulations. The oldest mean ram age of 2.76 years was reported in the COM subpopulation (Table 5).

**Table 5:** Mean ages and genetic trends of ewes, rams and adjusted lambs born for each subpopulation

	Subpopulations				
	CON	PRO	PROFOR	FOR	COM
Mean age (years of age)					
Ewes	3.54	3.97	3.97	3.97	3.49
Rams	2.61	2.51	2.51	1.00	2.76
Genetic trend^a^ (€ per year)					
Ewes	0	1.50	1.50	0.72	0.05
Rams	0	0.95	0.95	0.95	0.04
Adjusted lambs born	0	3.96	3.96	2.38	0.14

*CON* conservative breeders, *PRO* progressive breeders, *PROFOR* progressive foreign breeders, *FOR* foreign breeders, *COM* commercial farmers

^a^Genetic trend of the Irish €uro-star Replacement Index after adjustment for the age profile of each subpopulation (source: Sheep Ireland)

Genetic trends for rams, ewes and lambs, adjusted for the mean age of the subpopulation, were greatest for the PRO and PROFOR subpopulations. The genetic trend of rams in the FOR subpopulation, was equal to that of the PRO and PROFOR subpopulations. However, the genetic trend of ewes in the FOR subpopulation was inferior to that of PRO and PROFOR subpopulations (− €0.78), and therefore resulted in a lower overall genetic trend for lambs born (− €1.58) compared to the PRO or PROFOR subpopulations. The genetic trend for the COM subpopulation was positive for all groups of animals, albeit making little genetic progress (Table 5).

### Industry benefits

**Fig. 3:**
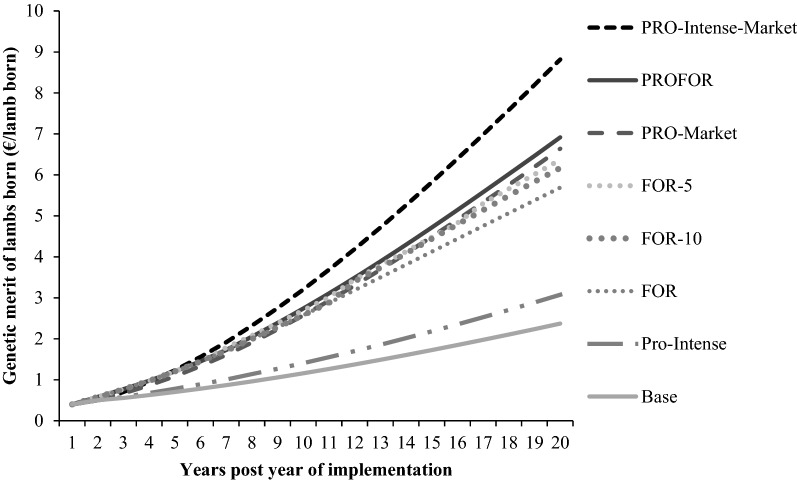
Genetic merit of commercial lambs from year 1 to 20 after implementation of each scenario. Scenarios investigated: ^‘^base scenario’ represents the current Irish industry; ‘PRO-intense scenario’ incorporates a reduction in the proportion of rams that are selected for mating from the top 40% to the top 20% and no shift in market share; ‘PRO-market scenario’ gradually shifts the market share of the CON subpopulation to the PRO subpopulation; ‘PRO-intense-market scenario’ includes a combination of a shifting market share towards the PRO subpopulation and a reduction in the proportion of rams that are selected for mating from the top 40% to the top 20%; ‘PROFOR scenario’ shifts the market share of the CON subpopulation between PRO, FOR and PROFOR; ‘FOR’, ‘FOR-5’ and ‘FOR-10’ scenarios includes the commercial subpopulation sourcing FOR sires directly from New Zealand once, every 5 years or every 10 years, respectively

**Fig. 4:**
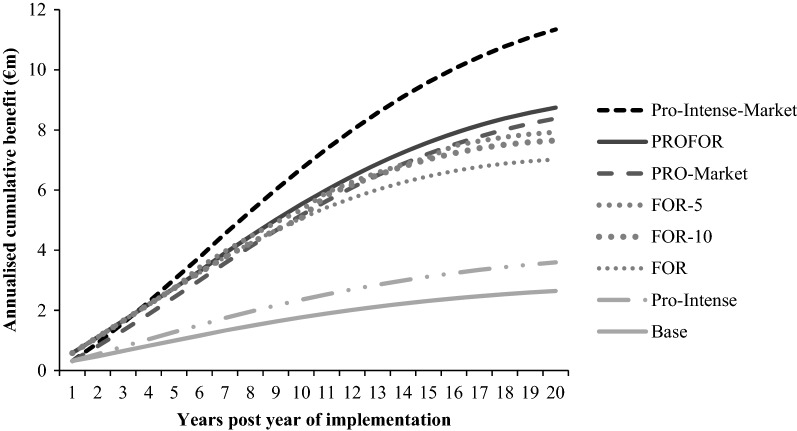
Annualised cumulative benefit from year 1 to 20 after implementation of each scenario. Scenarios investigated:’base scenario’ represents the current Irish industry; ‘PRO-intense scenario’ incorporates a reduction in the proportion of rams that are selected for mating from the top 40% to the top 20% and no shift in market share;‘PRO-market scenario’ gradually shifts the market share of the CON subpopulation to the PRO subpopulation; ‘PRO-intense-market scenario’ includes a combination of a shifting market share towards the PRO subpopulation and a reduction in the proportion of rams that are selected for mating from the top 40% to the top 20%; ‘PROFOR scenario’ shifts the market share of the CON subpopulation between PRO, FOR and PROFOR; ‘FOR’, ‘FOR-5’ and ‘FOR-10’ scenarios include the commercial subpopulation sourced FOR sires directly from New Zealand once, every 5 years or every 10 years, respectively

The change in the average genetic merit value and economic benefit for the commercial lambs born are presented in Table 6, and Figs. 3 and 4. The ranking of scenarios based on their economic benefit was the same as the ranking of genetic trends, with the exception of FOR-5, which surpassed PRO when presented as an annualised cumulative benefit (ACB) [€10.92 vs. €10.90 million (m) per year].

**Table 6:** Genetic merit and economic benefits of commercial lambs born in year 20 for each scenario

Scenario	Genetic merit	Economic benefit	
	Replacement index	PVB	ACB
	Euro per lamb born (€)	Millions of euro (€)	
Base	2.51	45.28	3.63
PRO-intense	3.44	60.65	4.87
PRO-market	7.09	135.84	10.90
PRO-intense-market	9.45	179.93	14.44
PROFOR	7.37	144.28	11.58
FOR	6.00	123.88	9.94
FOR-5	6.72	136.14	10.92
FOR-10	6.50	132.15	10.60

‘Base scenario’ represents the current Irish industry; ‘PRO-intense scenario’ incorporates a reduction in the proportion of rams that are selected for mating from the top 40% to the top 20% and no shift in market share; ‘PRO-market scenario’ gradually shifts the market share of the CON subpopulation to the PRO subpopulation; ‘PRO-intense-market scenario’ includes a combination of a shifting market share towards the PRO subpopulation and a reduction in the proportion of rams that are selected for mating from the top 40% to the top 20% ; ‘PROFOR scenario’ shifts the market share of the CON subpopulation between PRO, FOR and PROFOR; ‘FOR scenario’ the commercial subpopulation sourced FOR sires directly from New Zealand; FOR-5 and FOR-10 scenario the commercial subpopulation sourced FOR sires directly from New Zealand, however new shipments were imported every 5 or 10 years, respectively

PVB: Present value of benefits is an indicator of the total potential economic benefit gained due to the implementation of each scenario, while accounting for the change in the value of money over time

ACB: Annualised cumulative benefit is an indicator of the potential annual economic benefit gained due to the implementation of each scenario, while accounting for the change in the value of money over time

All scenarios resulted in an increase in the average genetic merit value of commercial lambs born and an increase in both the present value of benefits (PVB) and the ACB generated, over and above the base scenario, after 20 years of implementation. The PRO-intense-market scenario contributed the greatest genetic and economic benefits to the industry after 20 years and resulted in a 3.7- and 4.0-fold increase in the rate of genetic and economic gain above the base scenario, respectively (Table 6). The incorporation of foreign genetics through the PROFOR scenario resulted in an average genetic merit value for commercial lambs born in year 20 of €7.37. When the COM subpopulation sourced rams directly from FOR, using either the FOR, FOR-5 or FOR-10 scenarios, the average genetic merit value for the commercial lambs born were €6.00, €6.72 and €6.50, respectively. The long-term use of foreign genetics without the replenishment of new rams (i.e. FOR scenario) increased the rate of genetic gain by 2.4 times over the base scenario. However, both the genetic and economic benefits increased in line with the frequency of importation of new genetics. The importation of FOR genetics every 5 years (i.e. FOR-5 scenario) increased the rate of genetic gain by 2.7 times over the base scenario. However, regardless of the specific FOR scenario investigated (i.e. FOR, FOR-5 or FOR-10) both the genetic and economic benefits were always lower than those achieved through the increased use of progressive domestic genetics (i.e. PRO-market or PRO-intense-market scenarios).

## Discussion

With increasing interest in the incorporation of superior genetics within the agricultural industry, research must investigate the potential benefit of the use of foreign sires in terms of genetic gain and consequent economic benefit within domestic industries. Previous studies in the dairy and beef cattle industries [2, 3], using similar modelling techniques as those used in the current study, have shown that at least a two-fold increase in the average rate of genetic gain in a domestic industry can be achieved through the importation of foreign genetics. The establishment of INTERBULL and INTERBEEF have highlighted the frequent transfer of germplasm between countries, in the dairy and beef cattle industries [15]. Although, there is some evidence within the sheep industry of the transfer of germplasm between countries, this is mostly on a small scale [12, 16]. Through an Ireland-New Zealand case study, this simulation examined various breeding strategies that could be implemented within the sheep industry to maximise the potential genetic benefits that can be achieved from importing superior foreign sheep genetics for widespread use, in this case New Zealand genetics, into the domestic industry, in this case Ireland.

Modelling genetic gain within an industry is not a new concept [8], however, the steady state model approach, as previously used by Nicholas and Smith [17] does not account for the genetic time lag between the nucleus flocks making a breeding decision and its subsequent impact on the commercial population. Such genetic time-lags can be considerable and can be as long as 5 to 11 years between nucleus and commercial tiers [4, 17]. In the current study, the calculated genetic time-lag between the PRO subpopulation and the COM subpopulation was 9 years under the PRO-intense-market scenario. When a steady state model was used to simulate the PRO-intense-market scenario (therefore removing the genetic lag), the genetic and economic benefits to the industry were grossly overestimated with a PVB and an ACB of €560.8 m and €45.0 m, respectively, by year 20; three times greater than those generated by the deterministic model, which included the genetic time-lag. Therefore, including a genetic time-lag in the model was more realistic to what actually occurs within the population during that period.

Although a deterministic modelling approach was undertaken in the current study, alternative modelling approaches such as stochastic simulation models could also have been considered [18]. However as previously discussed by Matthews et al*.* [3], stochastic simulation models are deemed to be too complex for industries with multiple subpopulations. Deterministic models have been demonstrated to be the most suitable method for calculating gene flow modelling [1–3, 5], as these models can account for the genetic time-lag involved in transitioning from one breeding programme to another while at the same time being parameterised using actual industry data and demonstrating the genetic and economic benefits of implementing various scenarios on the industry, similar to the approach undertaken in the current study.

Within the present and other studies [2, 3], a breakdown of the proportion of ewes and rams of different ages within each subpopulation was incorporated into the model, to demonstrate its impact on genetic gain. In the current study, the genetic merit of lambs born increased annually, therefore retaining older animals will reduce the overall genetic merit for the entire population through a lag effect, which depends on the generation interval as previously shown by Bichard [4]. A previous study that investigated the potential gain achievable within the Danish Jersey cow herd as a result of using young bulls compared to progeny-tested bulls did not however incorporate the age structure of the national cow herd into the model [19], perhaps overlooking the national replacement rate and ability for genetics to flow through the population. If such an approach of evenly dividing the age structure within each subpopulation was incorporated in the present study, rather than using the actual age structure from industry data, the estimated benefit to the industry from the PRO-intense-market scenario would be reduced by €21.5 m after 20 years. Horton et al. [5] highlighted the need for sheep breeders to use younger rams for mating, in order to shorten the generation interval and thereby increase the rate at which superior genetics reach the commercial industry. These authors also demonstrated the possibility of changing the three-tiered breeding structure to a two-tiered structure, once the nucleus was large enough to supply directly commercial farmers [5], such as was undertaken in the base scenario of the current study. The use of new technologies such as genomic selection could play an important role in reducing the generation interval and thereby the genetic time-lag in sheep populations [20]. With increased accuracy of parentage recording and the availability of more reliable genetic indexes, through the use of genomic selection [1, 21], the sheep industry could be more inclined to use a higher proportion of younger rams, as is currently the case in the dairy and beef cattle industries [9, 22, 23].

Reducing the proportion of rams selected for mating within a subpopulation was of key importance in maximising the genetic gain that can be achieved by the Irish sheep industry. The PRO-intense-market scenario, by incorporating a reduction in the proportion of rams selected for mating from 40% to 20% and an annual shift in market share, achieved the greatest increase in genetic and economic benefits, at 377 and 397% above that generated by the base scenario, respectively. The importance of selection intensity on maximising the rates of genetic gain in the Australian dairy industry was highlighted by the study of Pryce et al. [24], where the rate of genetic improvement increased up to 231%, relative to the progeny test scenario. However, the importance of determining the likelihood of an increase in inbreeding in the future, as a result of the implementation of any new breeding strategies must also be considered [16]. In this study, particular attention to the levels of inbreeding would be required for the PROFOR scenario, where limited amounts of foreign semen are imported at various intervals. These genetics are used within the PROFOR subpopulation, which acts as a multiplier tier, to then supply rams to COM farmers from within the domestic environment, rather than FOR genetics being sourced from a larger pool abroad.

As previously discussed and confirmed by Santos et al*.* [14], foreign genetics often rank higher for genetic merit than domestic genetics for many industries. For this reason, a superiority value of €2 was set for all the scenarios that included the use of foreign genetics. When comparing the results generated from this model, the rankings of both the genetic and economic benefits were similar for all scenarios with the exception of the FOR-5 and the PRO-market scenarios. While the average genetic merit of COM lambs born in the FOR-5 scenario was less than the PRO-market at year 20, the ACB was greater for the FOR-5 scenario than the PRO scenario. This change could be attributed to the rapid initial boost in which lambs born in the FOR subpopulation when $Y=0$ had an average genetic merit value that was twice that of the lambs born in the PRO subpopulation (€6.05 vs. €3.01), as they benefited from the additional level of superiority. However, the long-term impacts of the FOR-5 scenario are hampered by the foreign population having a lower genetic trend than the domestic population; €0.18 vs. 0.38, for which the average genetic merit value of the lambs born within the FOR subpopulation was only €0.90 greater than the PRO subpopulation when $Y=20$. Gains plateaued from year 14 onwards.

However, the additional superiority of the FOR genetics (i.e. €2 greater than all domestic genetics) did not prove to be as beneficial as predicted. This could be explained by the frequency of importation of the FOR genetics and the modest annual shift in market share of 5% of the value in the previous year, away from conservative breeders towards progressive breeders modelled in the current study. In the FOR, FOR-5 and FOR-10 scenarios, the average genetic merit value of lambs born within the PRO and FOR subpopulations equalised during years 12, 15 and 17, respectively. In addition, the breeds of New Zealand sheep imported as part of [12] and used to calculate the regression coefficient between the $DOM index$ and $FOR index$ in the current study comprised terminal type breeds (i.e. Suffolk and Texel), which are similar to the ewe breed type in Ireland [25]. The importation of more maternal based breeds with a greater genetic trend could have been considered for the FOR scenarios but this would not reflect the breeding practices that are currently operated in Ireland and would therefore have required a dramatic change in the Irish breeding structure. Alternatively, if the superiority of the foreign genetics over the domestic genetics was increased to €4 (rather than €2 as investigated in the current study), the FOR genetics would be superior to the PRO genetics beyond year 20. The low foreign genetic trend (€0.18) used in the current study could be explained by the fact that the New Zealand selection pool available for importation to Ireland was severely reduced, as only live animals (no semen or embryos), with a Scrapie type 1 status can be imported at what amounts to a sizeable cost [26]. The potential may exist for the foreign genetic trend to surpass the domestic genetic trend, if semen and embryos were available to the domestic industry in the future. The time and costs associated with the importation of foreign genetics, the availability of fresh and/or frozen semen given the seasonal nature of the breeding season and the hiring of a registered veterinarian to carry out the laparoscopic artificial insemination procedure, which is a procedure that is currently used by only a small number of pedigree flocks in Ireland, all prove to be serious obstacles to overcome before the PROFOR, FOR, FOR-5 or FOR-10 scenarios can be implemented successfully in practice in Ireland.

Furthermore, the authors carried out a sensitivity analysis on the FOR-5 and FOR-10 scenarios to examine the potential impact of the importation of foreign genetics with a greater foreign genetic trend than that achieved by the New Zealand population within this particular case study. Results were expressed relative to the current genetic trend within the PRO subpopulation (i.e. €0.38), in order to demonstrate the difference in foreign genetic trend relative to the domestic trend. As previously mentioned, the PRO-intense-market scenario generated an additional €179.93 m for the industry (PVB), at an ACB of €14.44 m per year and increased the genetic merit of lambs born to €9.45 after 20 years of implementation. The genetic and economic benefits achieved within the adjusted FOR-5 and FOR-10 scenarios reached similar levels to those achieved by the PRO-intense-market scenario when the foreign genetic trend was increased to 2.5 and 3.5 times that of the domestic genetic trend, respectively. The adjusted FOR-5 and FOR-10 scenarios generated a PVB and an ACB of €188.21 m and €15.10 m, and of €184.62 m and €14.84 m, respectively. A significant increase in foreign genetic trend was required in order to generate gains equal to the PRO-intense-market scenario. This was attributed to the modest shift of 5% per year of the market share value in the previous year. An increased shift in market share away from conservative breeders at a rate of 10% each year as part of the FOR-5 scenario increased the PVB by €50.24 m, the ACB by €4.03 m, and the genetic merit of lambs born by €1.88 m after 20 years (results not presented). However, in a volatile industry where tradition is at the forefront within the domestic Irish industry and substantial genetic progress is only in recent years being achieved, 5% was a realistic but changeable parameter within the model.

Although the genetic trends within Ireland and New Zealand differ, breeding objectives within both countries are similar, as industries attempt to breed a robust ewe that is suitable in any grass-based production system. Previous work completed by Santos et al. [14] demonstrated the similarity of the breeding objectives between both countries, particularly the maternal indexes which are strongly correlated (0.86). While traits within the maternal indexes of each country have different weightings, a similar direction in response to selection is present for most traits including number of lambs born, survival and growth. Whereas the New Zealand maternal system has been shown to be compatible with that in Ireland, the variety of maternal breeds within both countries differ. Although Ireland’s sheep industry is more likely to make a more substantial genetic gain within the maternal index through the importation of maternal sheep breeds from New Zealand that benefit from a higher base maternal genetic index, this case study is based on the importation of high maternal genetic merit Texel and Suffolk ewes. Due to a variety of reasons including tradition and the fact that sheep are more environmentally adapted than cattle, it is likely that farmers would be more hesitant to import other unfamiliar foreign maternal breeds; such evidence of their poor uptake is apparent in the UK and Australian industries. The sheep industry is unlike the beef industry, which is hugely driven by importation of foreign genetics, and for which the vast majority of the genetic progress around the world over the last 40 years has developed due to importation of superior beef genetics. The US and France have been the main drivers of this due to their superior base genetic gain and, in turn, allowing other countries to import and therefore benefit from their genetic progress. To date, importation within the sheep industry has been somewhat limited. However, New Zealand has benefitted from the importation of foreign genetics in the past through the integration of alternative breeds such as Finn, East Friesian and Texel into the national breeding programme. Although integrated in relatively modest numbers, with the national maternal flock now comprised of approximately 20% Texel, 5% Finn and 1% East Friesian, their contribution has been high through the creation of variation and encouraging change within traditional practices. However, before foreign genetics are imported, countries must consider the initial genetic merit of the animals available and the genetic trend being achieved within the foreign country. If the domestic industry is already achieving genetic progress, the foreign genetic trend needs to be substantially greater than the domestic genetic trend. If little to no progress has been achieved to date within the domestic industry, then the importation of foreign genetics with a modest genetic index and genetic trend should be carefully considered as a potential method to quickly and effectively contribute to economic and genetic progress. Although only New Zealand FOR genetics were considered in the current study, other countries such as the UK and France have similar breeds and operate similar breeding objectives to Ireland, therefore these countries could also have been considered. However, results presented by Fitzmaurice [27] and Englishby et al*.* [28] highlight the consistent flow of both sheep and beef genetics from the UK into the Irish industry, proving that many UK bloodlines already exist in Ireland and therefore the importation of such genetics is unlikely to yield the same benefits as the superior New Zealand genetics.

In practice, realisation of this opportunity for industry benefits would depend on considerable change in the farmer ram buying behaviour. Experience in the beef industry in Ireland would suggest that a national development programme with quite rigorous genetic improvement requirements has been shown to be effective at changing farmer behaviour. For this reason, the authors recommend that the Department of Agriculture, Food and the Marine, in collaboration with Sheep Ireland [29] (i.e. the national body with the responsibility of operating Ireland’s sheep breeding database and developing Ireland’s sheep breeding strategies) should undertake the primary roles in the operation and application of the PRO-intense-market scenario. Implementing a voluntary programme where farmers are rewarded for the use of superior genetics could be considered. Similar programmes have been successfully implemented in Ireland in the past within the beef industry, including the Beef Data and Genomics Programme. This programme promoted the use of high genetic merit maternal bulls and resulted in an increase in the €uro-star replacement index of the national herd, which had remained relatively stagnant until its implementation [30]. However, within the sheep industry the use of superior genetics must be promoted to and insisted upon by COM farmers in order to shift demand, which could be triggered by the importation and use of foreign genetics, away from CON breeders, who select animals based on physical attributes rather than genetics and achieve no increase in their rates of genetic gain, yet hold a majority stake in market share. Therefore, an opportunity to implement a voluntary programme exists as the current phase of the Sheep Welfare Scheme [31] is coming close to its conclusion in 2020. As the next phase of the aforementioned scheme or an alternative programme is established, opportunity exists to incorporate the use of superior genetics as a requirement within a scheme. Furthermore, while significant potential exists to increase genetic and economic benefits within the Irish sheep industry, one cannot forget that the fundamental aspects of this model, including the use of genetic evaluations, the shift in market share away from conservative breeders, the shift in the proportion of rams that are selected for mating or the importation of superior foreign genetics could possibly be incorporated into many breeding programmes internationally in order to make substantial gains possible. This model could easily be adapted for use within other countries, for example, those involved with across-country genetic parameters such as INTERBEEF, with members including Australia, Sweden, United Kingdom, Italy, France, Germany, Switzerland, and South Africa, or countries that share germplasm.

## Conclusions

The findings of the current study demonstrate that it is possible to substantially increase the genetic and economic benefits to the domestic industry, without foreign sire contributions, but through the strategic use of domestic genetics. Essential to its success is a shift in the market share from conservative breeders towards progressive breeders. The use of foreign genetics may play a key role in triggering this shift. Deterministic modelling, which incorporated age proportions, genetic time-lags, the proportion of rams that are selected for mating, superiority of foreign genetics compared to domestic genetics, selection differentials and proportion of market share proved to generate realistic methods that could be incorporated into the national breeding programme in an attempt to increase genetic and economic benefits to the domestic industry. This model now provides a template for other industries to quantify their genetic and economic benefits as a result of foreign sire contributions, regardless of country or species.

## Supplementary Information

**Additional file 1.** Derivation of Eq. 1: $G_{{W,~Y = 0}}^{{Rams~born}} = G_{{W,~Y = 0}}^{{ewes}} + ~\Delta G_{{W,~Y \le 0}} ~\left( {\bar{X}_{{W,~Y = 0}}^{{rams}} + 2\cdot\bar{X}_{{W,~Y = 0}}^{{ewes}} } \right).$ The derivation of the equation used to calculate the genetic merit of rams born within a given subpopulation at year 0.

## Data Availability

The datasets used and/or analysed during the current study are available from Sheep Ireland [https://www.sheep.ie/wp/], Sheep Improvement Limited [https://www.sil.co.nz/] or from the corresponding author upon reasonable request.
