# Supplementary material for: Genetic and economic benefits of foreign sire contributions to a domestic sheep industry; including an Ireland-New Zealand case study
Source: Genet Sel Evol. 2021 Jan 6;53:5. doi: 10.1186/s12711-020-00594-y (PMC7789235; doi:10.1186/s12711-020-00594-y)
Supplement: Supplementary file 1 — Additional file 1. Derivation of Eq. 1: $G_{{W,~Y = 0}}^{{Rams~born}} = G_{{W,~Y = 0}}^{{ewes}} + ~\Delta G_{{W,~Y \le 0}} ~\left( {\bar{X}_{{W,~Y = 0}}^{{rams}} + 2\cdot\bar{X}_{{W,~Y = 0}}^{{ewes}} } \right).$GW,Y=0Ramsborn=GW,Y=0ewes+ΔGW,Y≤0X¯W,Y=0rams+2·X¯W,Y=0ewes. The derivation of the equation used to calculate the genetic merit of rams born within a given subpopulation at year 0. [file 12711_2020_594_MOESM1_ESM.docx]

**Additional files**

**Additional file 1**

Deriving equation 1: $G_{W, Y=0}^{Rams born}=G_{W, Y=0}^{ewes}+ {\Delta G}_{W, Y\leq0} \left( \bar{X}_{W, Y=0}^{rams}+2\cdot\bar{X}_{W, Y=0}^{ewes} \right)$

${MM}_{t}$= Genetic merit of mated males at time $t$

${FM}_{t}$= Genetic merit of mated females at time $t$

$P_{t}$= Genetic merit of progeny at time $t$

${SMB}_{t}$= Genetic merit of selected males born at time $t$

We assume a constant genetic trend △ across the four categories of animals above.

$\bar{X}_{m}$= Average age of $MM$ at mating

$\bar{X}_{f}$= Average age of $FM$ at mating

Progeny at time $t$ have the average genetic merit of their parents:

$P_{t}$=$\frac{{MM}_{t}}{2}$+ $\frac{{FM}_{t}}{2}$ [Equation 1]

Males mated $(MM)$ have the genetic merit of the selected males born $(SMB)$ but after a lag of $\bar{X}_{m}$ years,

i.e. ${MM}_{t}$=${SMB}_{t-\bar{X}_{m}}$ = ${SMB}_{t}$ - $\bar{X}_{m}\triangle$ [Equation 2]

Females mated $(FM)$ have the genetic merit of the progeny born $(P)$ but after a lag of $\bar{X}_{f}$ years,

i.e. ${FM}_{t}$=$P_{t-\bar{X}_{f}}$ = $P_{t}$ - $\bar{X}_{f}\triangle$ [Equation 3]

so if we rearrange equation 3 we get:

$P_{t}= {FM}_{t}+ \bar{X}_{f}\triangle$ [Equation 4]

Substituting equation 2 and equation 4 into equation 1 we get:

${FM}_{t}+ \bar{X}_{f}\triangle$ = $\frac{{SMB}_{t} - \bar{X}_{m}\triangle}{2}$ + $\frac{{FM}_{t}}{2}$ [Equation 5]

Rearranging equation 5 as follows gives:

$\frac{{SMB}_{t} - \bar{X}_{m}\triangle}{2}$ = ${FM}_{t}+ \bar{X}_{f}\triangle$ - $\frac{{FM}_{t}}{2}$

${SMB}_{t} - \bar{X}_{m}\triangle=2\left( {FM}_{t}+ \bar{X}_{f}\triangle\right)-$ ${FM}_{t}$

${SMB}_{t}=$2${FM}_{t}$ - ${FM}_{t}$ + 2$\bar{X}_{f}\triangle$ + $\bar{X}_{m}\triangle$

$${SMB}_{t}={FM}_{t}+ \triangle(2\bar{X}_{f}+ \bar{X}_{m})$$

which equates to equation 1 in the manuscript.
